# Supplementary material for: Desmoid Tumors in the Active Surveillance Era: Evaluation of Treatment Options and Pain Relief in a Single-Center Retrospective Analysis
Source: J Pers Med. 2023 Nov 27;13(12):1653. doi: 10.3390/jpm13121653 (PMC10744644; doi:10.3390/jpm13121653)
Supplement: Supplementary file 1 [file jpm-13-01653-s001.zip › jpm-2691088-supplementary.docx]

**Supplementary material**

**Supplementary Table S1**. Number of patients who reported painful symptoms at diagnosis and after the first treatment. ASG: patients under active surveillance; MG: patients receiving pharmacological treatment; SGPost12 patients who underwent surgical treatment after 2012; SGPre12 patients who underwent surgical treatment before 2012.

| Treatment group | Pain at diagnosis | Pain after the first treatment | | p-value |
| --- | --- | --- | --- | --- |
|  |  | No | Yes |  |
| SGPre12 | No | 21 | 0 | 0.07 |
|  | Yes | 5 | 2 |  |
| SGPost12 | No | 27 | 2 | 0.07 |
|  | Yes | 9 | 2 |  |
| MG | No | 8 | 0 | 0.0001 |
|  | Yes | 16 | 11 |  |
| ASG | No | 10 | 2 | 0.99 |
|  | Yes | 1 | 3 |  |
